# Perceptions and Attitudes of Egyptian Health Professionals and Policy-Makers towards Pharmaceutical Sales Representatives and Other Promotional Activities

**DOI:** 10.1371/journal.pone.0140457

**Published:** 2015-10-16

**Authors:** Susan Kamal, Christine Holmberg, Jean Russell, Tomasz Bochenek, Beata Tobiasz-Adamczyk, Christiane Fischer, Peter Tinnemann

**Affiliations:** 1 School of Health and Related Research, University of Sheffield, Sheffield, United Kingdom; 2 Berlin School of Public Health, Charité – Universitätsmedizin Berlin, Berlin, Germany; 3 Department of Drug Management, Institute of Public Health, Collegium Medicum, Jagiellonian University, Krakow, Poland; 4 Department of Epidemiology and Preventive Medicine, Institute of Public Health, Collegium Medicum, Jagiellonian University, Krakow, Poland; 5 MEZIS, mein essen zahl Ich selbst, Hamm, Germany; 6 Institute for Social Medicine, Epidemiology, and Health EconomicsCharité – Universitätsmedizin Berlin, Berlin, Germany; the University of Sydney, AUSTRALIA

## Abstract

**Background:**

Pharmaceutical promotion activities in low and middle-income countries are often neither regulated nor monitored. While Egypt has the highest population and per capita use of medicines in the Arab world, we know very little about pharmaceutical companies promotional activities in the country.

**Aim:**

To explore and analyze the perceptions of physicians towards promotional and marketing activities of pharmaceutical companies among physicians and pharmacists in Egypt.

**Methodology:**

Perspectives of different healthcare system stakeholders were explored through semi-structured, in-depth interviews conducted in 2014 in Cairo, Egypt. Interviewees were chosen via purposive sampling and snowball technique. Each interview was recorded and transcribed. Then qualitative, thematic analysis was conducted with the help of NVIVO software.

**Findings:**

The majority of physicians and pharmacists acknowledged exposure to pharmaceutical promotion. It was commonly believed that interaction with the pharmaceutical industry is necessary and both associated risks and benefits were acknowledged. The interviewed physicians considered themselves competent enough to minimize risks and maximize benefits to their prescribing habits. Views diverged on the extent and magnitude of the risks and benefits of pharmaceutical promotion, especially in regard to the influence on patients’ health.

**Conclusions:**

Pharmaceutical promotion in Egypt is intensely directed at prescribers and dispensers. Physicians, pharmacists and policymakers expressed little skepticism to the influence of promotion towards their individual prescribing. Raising awareness of the pitfalls of pharmaceutical promotion is necessary, especially among the less experienced physicians.

## Introduction

Private pharmaceutical companies are expected to maximize their profits; therefore they have an interest in influencing sales of medicines [1], for example by providing medical practitioners with incentives to increase medicine consumption [2].

But regulation of pharmaceutical promotion is needed because of the conflict between the profit-oriented pharmaceutical industry's legitimate business goals to maximize returns on investment and patients, health care providers and societal expectations to use medicine in the most rational way[3, 4]. Nevertheless, regulating their efforts to increase profit is challenging especially since pharmaceutical companies are considered relevant for innovation in medicine. [5].

Egypt, as the largest Arab middle-income country, with a population of about 85 million people and central to modern Middle Eastern politics, plays an influential role for pharmaceutical companies’ promotional strategies [6,7]. For example, approval for the manufacture and sale of medicines in Egypt is seen as an opener to the pharmaceutical markets of other Arab countries [8–10]. Egypt is the largest pharmaceutical manufacturer in the Middle East and North Africa region, holding 30% of the regional market share, [11]. From among 47 pharmaceutical producers active in Egypt, 37 belong to the public sector, 4 to the local private sector and 6 are multinational companies. Multinational and local private companies hold more than half of the total market share [11]. In 2009 the expenditure for pharmaceuticals in Egypt was EGP 21,000 million (US$ 3,559 million) in total and EGP 253 (US$ 42.89) per capita [12]. Demographic, epidemiological and social transitions increase medicine consumption, making Egypt a lucrative market for pharmaceutical companies.

The Egyptian Drug Authority (EDA) is the regulatory body for the safety and quality of pharmaceutical products, the conduct of pharmaceutical practice and the availability of high-quality medicines at affordable prices [13]. In 2011 the EDA established the Department of Marketing Materials and Media Monitoring, aiming to review and ensure the integrity of promotional materials and to regulate the activities of pharmaceutical companies’ scientific offices [14]. However, limited resources are devoted to this activity or if there is no active monitoring of pharmaceutical marketing activities. While Western concepts of “bribery” and “corruption” often do not map directly onto the social and cultural practices in low- and middle-income countries, it is assumed that substantial influence on the prescribing habits of medical practitioners might occur due to weak regulation and monitoring mechanisms [15].

In this study we aimed to analyze the perception of and attitudes towards the promotional and marketing activities of pharmaceutical companies aiming to influence physicians’ prescribing in Egypt, as viewed from the perspectives of different stakeholders of the healthcare system.

## Materials and Methods

In order to understand healthcare providers’ perspectives on pharmaceutical promotion and influence, a qualitative study was conducted, including semi-structured in-depth interviews with physicians, dentists, pharmacists and policymakers [16]. The interview guide was developed after reviewing interview guides of other published studies with similar goals. The interview was designed to allow respondents to discuss the questions in their own words and to evoke responses that were rich and explanatory in nature [17]. In order to obtain an in-depth understanding of how different healthcare providers work in Cairo, how they are targeted by the pharmaceutical industry and experience such approaches we chose a purposive sampling strategy [18]. The selection criteria aimed to recruit physicians with different durations of work experience, gender and workplace (public or private), as well as dentists (who are also prescribers of medicine in Egypt) and policymakers equally with different lengths of work experience, gender and work setting. These characteristics were chosen because we assumed that they influence their level of exposure to pharmaceutical promotion and how they respond to it.

Interviewees were initially identified through the first author’s network of personal and professional contacts. In each interview, the interviewees were asked to recommend further participants based on what types of interviewee characteristics we were still seeking. The interviewees were approached by telephone to explain the project and interview procedure. Those agreeing to be interviewed signed an informed consent form. An information sheet explaining the research project in English language was provided to each participant. Most Egyptian physicians and pharmacists have a high level of English language skills since the major part of their education is in English.

The interviews were conducted in person by the first author, who is an Egyptian pharmacist, in Cairo over a period of 4 weeks in July and August 2013. Most interviews were audio-recorded. Only three participants refused to give consent to recording and written notes were taken. The interviews were conducted in the Arabic language, the mother tongue of all study participants and the interviewer. Recordings or notes were translated into English and transcribed. The interviewees were then asked to verify the English language transcriptions to ensure that translations maintained the original meaning.

After fully transcribing the data, the text material was entered into the Computer Assisted Qualitative Data Analysis package “QSR NVIVO version 10” [19]. Thematic analysis was performed to identify themes or patterns in the data [20]. The thematic structure was developed from gathered materials deductively based on the interview guide. Resulting thematic structures were analyzed seeking commonalities, relationships and explanatory principles. The initial coding used 25 different themes covering five main areas: pharmaceutical sales representatives; gifts and morality towards the kind of gifts; prescribing habits and potential influence from the pharmaceutical industry; laws and legal awareness; and health policy recommendations. The first author identified and grouped the different themes together with the third author. There was no opposition between them in the identified themes. In addition, linguistic analysis was done.

Ethical approval for this study was granted by the School of Health and Related Research (ScHARR) of the University of Sheffield Ethics Committee (Sheffield, the UK). All collected data and information was stored on a password-protected computer and accessed only by the first author. Full names of participants were not recorded and within this article their initials were changed to ensure participants’ anonymity. Full confidentiality and anonymity of participants was maintained throughout.

## Results

The initial recruitment process started with three health professionals (one senior physician, one middle-ranking psychiatrist and one junior dentist). Participating pharmacists and policymakers were professional contacts of the first author. All individuals interviewed worked in urban Cairo and agreed voluntarily to be interviewed. (see Table 1).

**Table 1:** Characteristics of Interviewees.

Characteristics	n (%)
Physicians	19
Male Sex	18(94)
Age (years)*	44.9 (±12.6)
Prescriptions (per week)*	103 (± 90)
Representative visits (per week)*	24.3 (± 17.6)
Work experience (years)*	22.3 (± 13.4)
*Senior physician (>20 years)*	*7 (36*.*8)*
*Middle ranking physician (6–20 years)*	*8 (42)*
*Junior physician (1–5 years)*	*4 (21)*
Specialisation	
*Orthopaedics*	*8*
*Internal Medicine*	*2*
*Surgery*	*2*
*Psychiatry*	*2*
*Dermatology*	*2*
*Urology*	*2*
*Anaesthesiology*	*1*
Dentist	1
Female	1
Pharmacists	5
Male Sex	4(80)
Age (years)*	29 (± 2.5)
Pharmacy Owner	3 (60)
Pharmacy employee	2 (40)
Work experience (years)*	5.6 (± 1.5)
Policy makers	2
Central Pharmaceutical Regulatory Agency Employees	

*Represented as means ± standard deviation (SD)

While all physicians worked for public hospitals, senior and middle-ranking doctors additionally owned private practices. Participating pharmacists either owned the pharmacy or were employees in community pharmacies in affluent city areas. Junior doctors were considered those in specialist training in their first five years after graduating from medical school, middle-ranking doctors had between 6 and 20 years of experience in medical practise and senior doctors had more than 20 years of experience.

### Physicians and dentist (prescribers) attitudes towards marketing activities and sales representatives

When asked about their relationship and interaction with pharmaceutical companies, all physicians and the dentist were aware of the business-related aspects of pharmaceutical promotion. They described it as: *“It is a business relationship*” (H.A.); *“It is a cautious relationship based on mutual benefit*. *They offer some benefits e*.*g*. *by inviting you to attend conferences*, *symposia on medicines*. *Sometimes they give out gifts or free medical samples*. *So there is a benefit for the physician”* (A.H.); or *“Detailing is important*. *To be able to prescribe a medicine it is important that the rep visits me once or twice to remind me of the medicine”* (A.M.). All physicians agreed that it was a profitable business: *“We all know that the pharmaceutical business is more profitable than the arms trade”* (Y.A.).

The pharmaceutical companies’ gifts to physicians varied from office supplies, to cash and invitations to conferences or sponsored continuing medical education (CME) events. Physicians commonly accepted free samples of medicines as beneficial for patients, since they distributed them among the poor or charity institutions. One physician asserted: *“For an antibiotic*, *if you give the patient one box and ask him to buy another box to complete the course of treatment*, *you are guaranteed that the patient will buy the box for sure from this company*. *And you save the patient some money*, *so it is a great thing”* (A.M.).

Physicians and the dentist described sales representatives as usually young—in their 20s to early 30s, and that they visited them almost on a daily basis. More senior physicians judged sales representatives’ scientific knowledge as being rather insufficient. One of them commented: *“The majority of the reps do not really have a good understanding of the medicine they are promoting and just say some sentences they know by heart”* (A.A.). Quoting physician H.M., the sales reps differ according to their educational background: *“Some have no clue and some have good information*. *It depends if they are pharmacy graduates or not*. *If they are*, *then they are good*, *other graduates just know some sentences by heart and do not have a good understanding of medical issues*.*”* Others observed differences in sales representatives’ knowledge depending on their employer: *“Their knowledge varies according to where they work*. *If it is a multinational company*, *they have a good knowledge of medical issues*. *The ones that work for local companies don’t*. *(…) For the local ones the level is very bad and it leads to lies*. *They do not add references*. *Like when they say the medicine has an efficacy of 80% there is no reference to this*. *The multinationals have a good level*, *however”* (Z.A.). Opinions on the quality of information presented verbally or through published materials were also probed. The interviewees felt that information often favored a particular company’s products. Usually they felt that they were not offered clinical trial reports or other scientific publications, unless they asked for them specifically. Quoting the dentist: *“Yeah*, *I think it is a bit misleading*, *the information they give*. *They are trying to sell a product*, *so they overrate it*. *The information is not wrong but they only talk about the positive things”* (N.I.). Physician E.U. said: *“I don’t think they write any wrong information*, *they only show the benefits in big font and colors*, *but the drawbacks are not listed*.*”*. Older and more experienced physicians tended to be more critical towards sales representatives’ knowledge, assuming that better quality information was delivered by sales representatives trained in pharmacy or those employed by multinational companies. Younger physicians assumed more often that pharmaceutical companies provide unbiased scientific knowledge and were less critical of the information provided.

Senior physicians stated that they did not have time to see sales representatives –one of them saying: *“A medical rep only spends a couple of minutes with me as I do not have time to listen to all that he has to say*. *I will not let in reps and leave my patients waiting outside”* (H.G.). Junior physicians, on the other hand, tended to assess the sales representatives’ medical knowledge differently. One described it as: *“When it comes to product knowledge*, *they have a good knowledge and they always answer when we ask questions*. *I would say they have high medical expertise”* (G.I.). Another junior physician stated: *“They have the upper hand in the beginning* [meaning of their career]) *because we do not know much about pharmacology”* (W.A.).

Senior physicians tended to describe the pharmaceutical industry sponsored conferences and continuing medical education (CME) events as important, whereas junior physicians were more interested in obtaining scientific articles and attended sponsored seminars less often. One of the senior physicians said: *“This CME is really important*. *It is good that they offer good education to junior medical staff to give them better opportunities to help them deliver a better service to the patients*. *It is important to employ promotion in a good way”* (A.H.). Physician O.H. commented on sponsoring attendance at conferences: *“Being sponsored at conferences or invited to them is good because you get good updates*. *Sometimes they hold them at a resort*, *which is nice*, *and then you can get time off”*.

When physicians were asked about their opinion on what constituted ethical promotion methods or acceptable gifts, they considered promotion involving scientific content and adding to the physician’s knowledge in general as ethical. While whatever could harm patients was considered as unethical. The Arabic expression for “bribery” was used quite often to refer to unethical promotion. A senior physician said: “*At the conferences held at resorts or the stand-alone events as they call them* … *there is very little scientific material compared to leisure*. *So it comes under bribery more than promotion” (Z*.*A*.). A junior physician added: *“What’s ethical is when the physician prescribes the medicine because he is convinced*. *What’s unethical is when they prescribe it in return for something” (Y*.*S*.*)*.

When asked to comment on how promotion might affect patient welfare, physicians gave conflicting views. Some believed that they could accept gifts for the benefit of patients: *“We sometimes take a donation for the hospital*. *Or with medical devices*, *say*, *I request five devices and I get the sixth for free*. *This is beneficial to the patien*.*”* (I.A.). Others believed that promotion might have a negative effect: *“If there is a cheaper medicine and the physician prescribes the more expensive one just to please the pharma company*, *this affects the patient negatively*” (H.A.). Some physicians believed that in general medicines were effective, or at least as soon as they had been approved to be marketed.

### Prescribers' beliefs about influences on prescribing decisions

When asked to describe the sources of information used in making prescribing decisions, physicians mostly referred to earlier medical studies. Further sources of information included scientific conferences, textbooks, scientific journals and individual experiences with pharmacological treatment. Some junior physicians reported following university staff prescription habits; others mentioned using the freely available information on the Internet.

Senior-level physicians did not feel influenced by pharmaceutical promotion in their prescription habits: *“I prescribe the most effective medicine*, *taking price into consideration*, *the one that does not have a lot of side effects”* (H.A.). Others commented on the influence of gifts: *“I accept the gifts but I am not necessarily influenced by them”* (E.U.).

The receiving of gifts was also an important topic in the interviews. For example, one physician described how he used the pens that were given to him as gifts but contended that they were not a part of his treatment decision-making: *“I get pens*. *It doesn’t affect me; I barely notice the names on the pens*. *Here in my clinic I have 20 pens with 20 names lying in front of me*. *I use them one by one until they are finished*. *They are just like regular pens for me”* (A.M.).

Interestingly, another important theme in the interviews was the influence on other physicians by pharmaceutical marketing. *“Some physicians prescribe when they expect something; others just prescribe because the medicine is good for the patient”* (N.S.). Another described it as: *“Physicians are offered gifts by some companies to prescribe their medication in return*. *And this is something all companies do*, *not one or two”* (A.M.).

*Some physicians ask for an air conditioner or to be sent on a trip to Turkey and this happens with physicians in the same building as my clinic*. *But I have never requested this”* (K.H.).

Some physicians also referred to the relationship between individual physician’s medical expertise and likeliness to be influenced by promotion. According to one physician: *“The physician who has no scientific background is more susceptible to be influenced by the pharma companies*. *If he doesn’t study and attend conferences*, *then the simplest thing will influence him*. *If you give him a pen with the medicine’s name on it he will prescribe it*. *If he is up to date and knowledgeable*, *even if they send him to a conference abroad*, *he will not be so influenced”* (O.M.).

Finally it was mentioned that adjuvants and non-prescription medicines were more easily overprescribed than prescription medicines. The dentist reported that while training with one senior dentist she noticed her being influenced by a sales representative’s visit: *“Yes I saw it happen in front of my eyes*. *The dentist saw a patient immediately after a rep had left the office mentioning one mouthwash*, *and she recommended that the patient should buy this specific mouthwash even though in my opinion it was not necessary for the patient at all”* (N.I.).

Some physicians reported fierce reactions to being subjected to influences: *“Sometimes they offer financial benefits if you*, *for example*, *prescribe 20 boxes per month*. *In this case*, *I kick the rep out of the clinic*. *They tried once or twice but when they found out I do not play this game*, *no one came near me afterwards”* (O.M.).

Others described the incentives provided by the pharmaceutical industry: *“To give you an example*, *in the cancer institute*, *any new physician who is offered a new job*, *arrives in a very poor financial state*. *After a little while*, *you see him driving a Mercedes*, *his wife a BMW and he travels abroad on holidays for to five times a year*. *Treatment costs around 75*,*000 Egyptian pounds and it is all paid by the government*. *This is a known syndrome that happens with expensive medicines”* (B.A.).

### The pharmacists’ attitudes towards pharmaceutical promotion and pharmaceutical sales representatives

Pharmacists had various attitudes towards pharmaceutical promotion. One pharmacist (M.R.) characterized pharmaceutical promotion as follows: *“The reps describe the medicine and its mechanism of action and they quite often compare it with their competitors*. *I do not think this is a good thing as they promote their medicine and make the competitor look bad*. *If there is a patient at the pharmacy at that time*, *they could be influenced by this”* (M.R.). Another pharmacist added: *“In Egypt*, *promotion is about financial promotion*, *not ethical promotion*. *So it is about how much profit you make*, *not whether this is an effective medicine*. *Is the medicine being promoted the best in the market*? *I doubt it”* (A.A.).

The pharmacists further reported that they were aware of incentives given to physicians to influence their prescription habits. They stated that they were even sometimes able to recognize this influence in an individual physician’s prescribing, e.g. when a physician added an unnecessary adjuvant to his prescriptions. As one pharmacist described it: *“As pharmacists*, *we do not receive personal gifts*, *only in the form of bonuses and discounts*. *For physicians*, *however*, *they need to give material incentives*, *a personal service*, *such as travel to a conference abroad*, *in order to motivate them to prescribe”* (P.H.). The interviewed pharmacists stated that they usually did not advise the patient against the physician’s prescription, regardless of their personal opinion.

### Prescribers and pharmacists awareness and opinions towards regulation of promotion

The interviewees were asked about their knowledge concerning laws or regulations to control and monitor pharmaceutical promotion, or whether they could suggest legislative solutions. Their answers suggest either a lack of knowledge, or the rather limited effectiveness, of existing legal regulations. One senior physician said: “*I do not think we have any laws*. *I only know of international laws*. *They are applied in multinational companies*, *in local companies not at all”* (H.A.). Another one described the situation rather sarcastically: *“The market now is a joke*, *there are no regulations*. *When there are seven types of the same medicine on the market*, *and you do not know the efficacy of them*, *this is not regulated*. *Most of the medicines are passed under the Ministry of Health as a food supplement*. *And this is not right”* (H.G.). Others believed that the situation was improving, for example A.H. said: *“The regulations are OK*. *There was a lot of corruption in the past 15 years*. *It was very open; spouses and family were allowed on trips without any scientific aims”*.

The interviewees claimed that there was a need for more monitoring and regulation of pharmaceutical promotion, arguing that patients’ health and welfare were at stake. They called for more monitoring, control and transparency of information on medicine efficacy: “*We need transparency of the efficacy of a medicine*. *And especially for chemotherapy patients*, *we need to verify if the prescribed medicine is effective for their condition or not”* (B.A.). Another physician added: *“There should be a restricted system for the medical reps and they need to be supervised*. *They shouldn’t offer money to physicians or send them to conferences to make them prescribe the medicine they suggest*. *There should be a regulatory body that monitors this*, *supervised by the Ministry of Health”* (M.A.). Regarding sponsored lectures at conferences, one physician suggested: *“If I am a speaker I should admit whether the company have given me any money*, *materials or research funds to deliver my presentation*. *Then the audience can judge whether my talk is biased or not”* (H.A.).

Pharmacists’ opinions on the importance of pharmaceutical regulations were rather equivocal. *“In general*, *pharmacists deal with customers*, *not patients*. *So the whole idea of serving patients does not exist*, *it is about making profit from this customer*. *There should be regulations concerning all the incentives given to physicians*. *Sometimes pharmacies hire sales people to sell medicine instead of pharmacists*, *and they sell narcotics to people”* (R.A.). Another pharmacist said: *“It is a square of four sides*, *the pharmacist*, *the patient*, *the physician and the medical rep*. *The four sides have to meet*. *Those four sides have to communicate to end up benefiting the patient”* (M.E.).

### Policy Makers’ awareness and opinions towards regulation of promotion

The interviewed policymakers stated that the government’s monitoring of pharmaceutical promotion in Egypt related to printed marketing materials. Before starting disseminating any materials, pharmaceutical companies are required to obtain approval from the Central Pharmaceutical Agency. However, the policymakers mentioned that nowadays the majority of pharmaceutical companies use digital methods of dissemination instead of printed materials and they highlighted the lack of regulations to control digital marketing materials. In addition, it is noted that if a company does not get any approval for their printed materials, there are no sanctions in place.

Policymakers stated that direct-to-consumer advertising of prescription medicines on national public television is prohibited. However, they added that satellite or non-state-owned television channels allowed advertising of prescription medicines and it was difficult to enforce regulations there.

They also believed that there was a strong need to enforce punitive laws on companies that do not follow Ministry of Health or WHO ethical criteria for medicinal drug promotion guidelines. Quoting one of them: *“If the fine for not following the guidelines is only 5 Egyptian pounds*, *no company is going to follow the guidelines; they have no reason to do so”* (M.F.).

## Discussion

In this study we investigated the views of those targeted by pharmaceutical promotion on promotional practices and how this may influence their healthcare provision. All were aware of promotional practices and had examples of influence on prescription practices that they had witnessed or discussed. In particular, pharmacists stated that there were certain cues on prescriptions that made them assume the influence of a pharmaceutical company. The types of pharmaceutical activities to influence prescription habits mirrored those in other countries, including the visit of sales representatives and the invitation to education sessions. None of the interviewees considered themselves to be influenced, and some were appalled by the provision of financial incentives to force a particular prescription habit on physicians.

The interviewed physicians reported not to question accepting gifts of little or no financial value but were more often opposed to receiving high-value gifts, describing them as bribes. We found that some considered promotional gifts with a medicine’s name on it to be helpful as a reminder of that name, which in fact seemed to be concordant with the pharmaceutical industry’s intentions. Physicians who acknowledged accepting gifts did so probably because receiving gifts from pharmaceutical companies was considered a common and acceptable practice in Egypt and elsewhere.

Compared to any other industry the pharmaceutical industry is ranked as having the highest return on investment [21]. Since pharmaceutical companies invest billions in marketing, targeting health professionals is expected to affect treatment decisions and prescribing habits [22]. Egyptian physicians were aware that pharmaceutical companies’ sales representatives are their direct contact with the industry. Although the reported visit frequency varied according to the physicians’ seniority in our rather selective and small sample this should be interpreted with caution. But in comparison, the reported visit frequency was higher than those reported for Turkey, Tunisia and Libya and Canada [23–26]. In Germany, most physicians were reported to be visited once a week and a minority of them every day [27].

Evidence from marketing research shows physicians’ sensitivity to pharmaceutical promotion and a direct relation between sales reps calls or detailing and sales [28]. In fact, not only do pharmaceutical sales representatives provide information favoring their products, they are also trained to spin physicians’ objections around and turn them into a positive selling point [29].

Awareness of the benefits and harms of medicines is essential in order to enable their rational use. Rational prescribing is not possible based on promotional materials’ information only, as exposure to promotion was found to be associated with lower prescribing quality, more frequent prescribing and higher prescribing costs [30]. In general, pharmaceutical companies’ promotional materials showed a serious lack of information on medicines’ harmful effects [31], suppressed unfavorable clinical trial results [32] and reps rarely mentioned serious adverse events [33]. In light of such findings our study results could be considered worrisome. The interviewed Egyptian physicians admitted that information provided to them by the industry often favored a particular company’s products over their competitors’ and that they only received independent original information on clinical trials if they asked for them specifically. Such practices have also been shown in a study on Libyan physicians who similarly reported that provision of drug information by pharmaceutical sales representatives was incomplete and often biased [34].

Another problem constitutes the pharmaceutical companies role in continued medical education (CME) of physicians and pharmacists. [35]. The interviewed Egyptian physicians judged industry-sponsored CME events to be of high quality, but considered the juxtaposition of information and promotion as uncomfortable. We would support a call for a greater role of independent postgraduate organizations in CME [36]. While the interviewed physicians reported awareness of the business relationship with pharmaceutical companies and acknowledged its potential harmful effects, they repeatedly declared that they individually were not influenced by gifts, whereas their colleagues were. Similar findings were also reported elsewhere [22]. The physicians interviewed claimed that small gifts cannot influence them. However, even accepting such gifts may have an influence on changing physicians’ attitudes and lowering their defenses against pharmaceutical marketing [37, 38]. The interviewed physicians were at large not aware of the potential effects of free medicine samples may have. While the interviewed physicians donated free medicine samples to patients in disadvantaged socioeconomic conditions, the availability of samples increases prescribing of the same brands [39], causes physicians to adopt new drugs more quickly [40] and shifts prescribing decisions towards less appropriate choices [41]. In the light of such findings, the very limited regulation and oversight in Egypt of pharmaceutical promotion to physicians is worrisome [42]. While a few companies, mostly multinationals, have released ethical codes of conduct, the absence of legislative regulations for promotion in low- and middle-income countries has potentially bad effects on individual patients’ health and the health system [23]. The interviewed individuals did only have limited knowledge about existing laws in Egypt. Thus, to enhance regulation and its visibility in Egypt it should be based on the WHO Ethical Criteria for Medicine Drug Promotion, especially since the WHO guidelines already provide principles that could be used to develop national legislation [43]. If the implementation of new regulations were to be accompanied by providing physicians with easily accessible, unbiased information on pharmaceuticals, obtainable from sources maintained independently of the pharmaceutical industry, one of the biggest issues of our interviewees (i.e. the inaccuracy of the presented information material) would be cared for.

### How pharmacists are influenced

Compared to physician–industry interactions, the interactions between pharmacists and industry are less commonly researched [44] Considerable attention is paid to individual pharmacists with influential roles, e.g. clinical specialists, drug information pharmacists, pharmacy and therapeutics committee members, clinical coordinators, and especially so called “thought leaders,” whose expertise is regarded to significantly influence clinicians and impact therapeutic decisions [45].

In our interviews, pharmacists working in privately owned pharmacies appear to have a pragmatic attitude towards pharmaceutical promotion. This is interpreted as giving lower relevance to medicine samples or personal experience and probably more weight to professional recommendation, textbooks or academic journals [46]. Probably since pharmacy schools tend to provide more teachings about pharmaceutical promotion compared to medical schools [47] and the amount of teaching on ethics and handling drug promotion has increased over recent years [48]. It was also found that pharmacists were more often taught on the ethics of drug promotion than physicians and showed a higher level of skepticism towards pharmaceutical marketing than physicians [48].

### Study limitations

In this study we obtained information from various stakeholders, and explored in-depth how those were involved in prescribing and distributing medicines while being under the influence of pharmaceutical industry’s promotional activities. These results are exploratory due to the limited and selected sample, and that we relied on self-report on prescribing habits and acceptance of gifts, rather than direct observation. In order to get a deeper understanding on how sales persons and small gifts may have an influence on everyday health care practices and how the free medications are used it would be of importance to conduct an observational study.

## Conclusions

In Egypt the pharmaceutical industry undertakes a wide array of marketing activities, including detailing, conference invitations, gifts and financial incentives. Whilst some physicians acknowledged the influence of pharmaceutical promotion on their individual prescribing, others believed they were immune to influence that would harm the patient, but considered their colleagues vulnerable to it. Pharmacists appeared more aware of pharmaceutical promotion’s impact on medicine prescribing, but felt helpless to interfere with doctor’s prescription habits influenced by pharmaceutical marketing. While policymakers suggested a need for more transparency, punitive laws and regulations to control pharmaceutical promotion in Egypt, the existing lack of up-to-date regulations governing pharmaceutical promotion and unregulated and poorly controlled industry-supplied information on medicines require more guidelines to govern the marketing activities of pharmaceutical companies in Egypt, given their potential to harm patients and the healthcare system as a whole.
